# Nobiletin and Derivatives: Functional Compounds from Citrus Fruit Peel for Colon Cancer Chemoprevention

**DOI:** 10.3390/cancers11060867

**Published:** 2019-06-21

**Authors:** Joanna Xuan Hui Goh, Loh Teng-Hern Tan, Joo Kheng Goh, Kok Gan Chan, Priyia Pusparajah, Learn-Han Lee, Bey-Hing Goh

**Affiliations:** 1 Biofunctional Molecule Exploratory (BMEX) Research Group, School of Pharmacy, Monash University Malaysia, Bandar Sunway 47500, Selangor Darul Ehsan, Malaysia; jxgoh2@student.monash.edu; 2 Novel Bacteria and Drug Discovery (NBDD) Research Group, Microbiome and Bioresource Research Strength, Jeffrey Cheah School of Medicine and Health Sciences, Monash University Malaysia, Bandar Sunway 47500, Selangor Darul Ehsan, Malaysia; Loh.Tan@monash.edu (L.T.-H.T.); lee.learn.han@monash.edu (L.-H.L.); 3 Institute of Biomedical and Pharmaceutical Sciences, Guangdong University of Technology, Guangzhou 510006, China; 4 School of Science, Monash University Malaysia, Jalan Lagoon Selatan, Bandar Sunway 47500, Selangor Darul Ehsan, Malaysia; goh.joo.kheng@monash.edu; 5 Division of Genetics and Molecular Biology, Institute of Biological Sciences, Faculty of Science, University of Malaya, Kuala Lumpur 50603, Malaysia; 6 International Genome Centre, Jiangsu University, Zhenjiang 212013, China; 7 Medical Health and Translational Research Group, Jeffrey Cheah School of Medicine and Health Sciences, Monash University Malaysia, Bandar Sunway 47500, Selangor, Malaysia; priyia.pusparajah@monash.edu; 8 Asian Centre for Evidence Synthesis in Population, Implementation and Clinical Outcomes (PICO), Health and Well-being Cluster, Global Asia in the 21st Century (GA21) Platform, Monash University Malaysia, Bandar Sunway 47500, Malaysia

**Keywords:** nobiletin, colorectal cancer, chemoprevention, bioactivities

## Abstract

The search for effective methods of cancer treatment and prevention has been a continuous effort since the disease was discovered. Recently, there has been increasing interest in exploring plants and fruits for molecules that may have potential as either adjuvants or as chemopreventive agents against cancer. One of the promising compounds under extensive research is nobiletin (NOB), a polymethoxyflavone (PMF) extracted exclusively from citrus peel. Not only does nobiletin itself exhibit anti-cancer properties, but its derivatives are also promising chemopreventive agents; examples of derivatives with anti-cancer activity include 3′-demethylnobiletin (3′-DMN), 4′-demethylnobiletin (4′-DMN), 3′,4′-didemethylnobiletin (3′,4′-DMN) and 5-demethylnobiletin (5-DMN). In vitro studies have demonstrated differential efficacies and mechanisms of NOB and its derivatives in inhibiting and killing of colon cancer cells. The chemopreventive potential of NOB has also been well demonstrated in several in vivo colon carcinogenesis animal models. NOB and its derivatives target multiple pathways in cancer progression and inhibit several of the hallmark features of colorectal cancer (CRC) pathophysiology, including arresting the cell cycle, inhibiting cell proliferation, inducing apoptosis, preventing tumour formation, reducing inflammatory effects and limiting angiogenesis. However, these substances have low oral bioavailability that limits their clinical utility, hence there have been numerous efforts exploring better drug delivery strategies for NOB and these are part of this review. We also reviewed data related to patents involving NOB to illustrate the extensiveness of each research area and its direction of commercialisation. Furthermore, this review also provides suggested directions for future research to advance NOB as the next promising candidate in CRC chemoprevention.

## 1. Introduction

Colorectal cancer (CRC) is the third most prevalent cancer reported in both men and women, ranking just after prostate or breast cancer and lung cancer [1]. Although in many cases there is no readily apparent cause of CRC, a number of factors have been found to be closely associated with this malignancy including gender, age, genetic predisposition, lifestyle, diet or as a complication from other diseases such as inflammatory bowel disease (IBD). Statistics showed that the death rate from CRC is 40% higher in males as compared to females, with the prevalence increasing with age, especially above 50 years old; however, there is a new and worrying trend of increasing incidence of colorectal cancer in the age group younger than 50, which, while slight, is still worrying [2,3].

The incidence of CRC has reduced as modern screening strategies have enabled much earlier detection of potentially malignant lesions, allowing for early intervention such as surgical excision of adenoma before it undergoes malignant transformation [4,5]. Although there has been a reduction, the high number of cases remains a major concern and the search for new and better treatments for CRC has been a key focus in pharmacological research. Standard therapy for cancer typically involves the triple regimen of surgery, chemotherapy, and radiation treatment. Efforts in exploring and developing new treatments are very much needed due to the limitations of the current treatment regimen—ranging from side effects, to complications and the development of drug resistance.

Researchers are attempting to explore multiple avenues for novel leads as anti-cancer agents with an increasing trend to focus on natural sources like plants and fruits [6,7,8]. However, while it is key to find new treatments to existing cancers, a crucial aspect that is also being explored is prevention of cancerous growths; in particular, this would be of benefit for those at risk due to the various factors outlined earlier. One of the effective strategies to control cancer is chemoprevention, which is defined as the use of a natural or synthetic agent to reverse, inhibit, or prevent the progression of cancer [9].

Plants and fruits are often part of a diet recommended to prevent various illnesses including cancer [10]. These beneficial properties may be from the chemicals they contain as well as their metabolites which enter our alimentary canal and eventually end up in our colon and rectum. If the compounds responsible can be isolated and purified for use as a treatment, this may be a milestone in new cancer therapies and prophylaxis. While an extensive review of polyphenols like apigenin and luteolin on anti-colorectal cancer effect can readily be found [11], this study highlights the potential chemopreventive effect on CRC of another flavonoid, namely nobiletin (NOB).

NOB, a polymethoxyflavone (PMF), is likely named after *Citrus nobilis*. This compound is one of the most ubiquitous flavones that can be isolated exclusively from the peel of citrus fruits [12]. Besides CRC, there is concurrently ongoing research looking into the effect of NOB on other types of cancers such as breast cancer [13,14], ovarian cancer [15], gastric cancer [16,17], lung cancer [18,19], liver cancer [20] and bone cancer [21]. There are also recent studies attesting to the benefits of NOB in anti-neurodegeneration [22,23], anti-diabetes [24], anti-obesity [25,26,27], antimicrobial [28], anti-allergy [29] and anti-inflammatory effects [30,31]. There are also a number of articles that support claims purporting to the role of NOB in reducing the risk of cardiovascular diseases [32,33] and osteoporosis [34,35].

Interestingly, this compound can be metabolised into a number of metabolites which also show significant anti-cancer effects. There are several recent reviews on the bioactivities of these citrus PMF [36] as well as the potential chemopreventive abilities of these PMFs toward cancers in general [37]. This review paper aims to gather the results of the in vivo and in vitro studies done in recent years and compile various molecular pathways by which the compound NOB and its derivatives act in CRC prevention which will in turn help to facilitate future research that targets these specific mechanisms.

## 2. Research Methodology

The main focus was to search for all relevant primary research papers published which looked into the use of NOB and its derivatives as a chemopreventive agent for CRC. A systematic search was performed to identify published literature on the chemopreventive potentials of NOB against CRC using Google Scholar. For studies published in foreign languages like Chinese, Japanese or Korean, an attempt was made to locate the translated version. The search strategy was performed using keywords ‘nobiletin’ and ‘colorectal cancer’ to locate relevant papers. This was also supplemented with keyword search of the terms ‘cancer statistics’, ‘colorectal cancer’, ‘colon cancer’, ‘metabolites’, ‘synergism’, ‘biotransformation’, ‘mechanism’, ‘apoptosis’, ‘anti-inflammatory’, ‘inflammation’, ‘cell proliferation’, ‘cell cycle arrest’, ‘metastasis’, ‘tumour’, ‘angiogenesis’, ‘absorption’, ‘metabolism’, ‘toxicity’, ‘distribution’, ‘elimination’, ‘solubility’ and ‘delivery’ combined using Boolean operators with ‘nobiletin’. PubChem and EMBASE were used as alternatives to ensure inclusion of all relevant papers while SciFinder database was mainly used to locate patents related to NOB. The reference lists of relevant articles collected were also screened for additional studies to be included in the review.

## 3. Nobiletin and Its Derivatives

The compound nobiletin (NOB) can be extracted exclusively from citrus fruits, namely mandarin oranges (*Citrus reticulate*), sweet oranges or Valencia oranges (*Citrus sinesis*), Miaray mandarins (*Citrus miaray*) [38], flat lemons or Hayata (*Citrus depressa*) [39,40], tangerines (*Citrus tangerine),* bitter oranges (*Citrus aurantium)* [12], Unshu Mikans or Satsuma mandarins (*Citrus Unshiu arnicia indica*) [41,42], Cleopatra mandarins (*Citrus reshni*) [43], mandarin oranges (*Citrus tachibana*), Koji Oranges (*Citrus leiocarpa*), Natsu Mikans (*Citrus tardiva*), Jimikan (*Citrus succosa*), Kinokuni Mandarins (*Citrus Kinokuni*), Fukushu (*Citrus erythrosa*), Sunkat (*Citrus sunki*) and hybrids of the mandarin orange with pomelo (*Citrus deliciosa*) [44]. *Citrus tangerine* was reported to contain the highest content of NOB, approximately five times of that in *Citrus sinesis* [45].

PMF can be isolated from orange peel through different types of chemical extraction processes, for example, the supercritical fluid extraction, microwave assisted extraction [46] and Soxhlet method, which is capable of extracting large sample volumes [43]. Through the supercritical fluid extraction process, the supercritical fluid extractor is used to process the orange peel grinds that have been freeze-dried. Then, the extract is further treated with carbon dioxide and ethanol to concentrate the bioactive compound [47]. A special method to improve NOB yield through the supercritical fluid extraction method is currently patented in Korea [48]. It was found that the maximal yield of NOB occurs at a temperature of 80 °C and pressure of 30 MPa with an optimum sample particle size of 375 μm [40].

**Figure 1:**
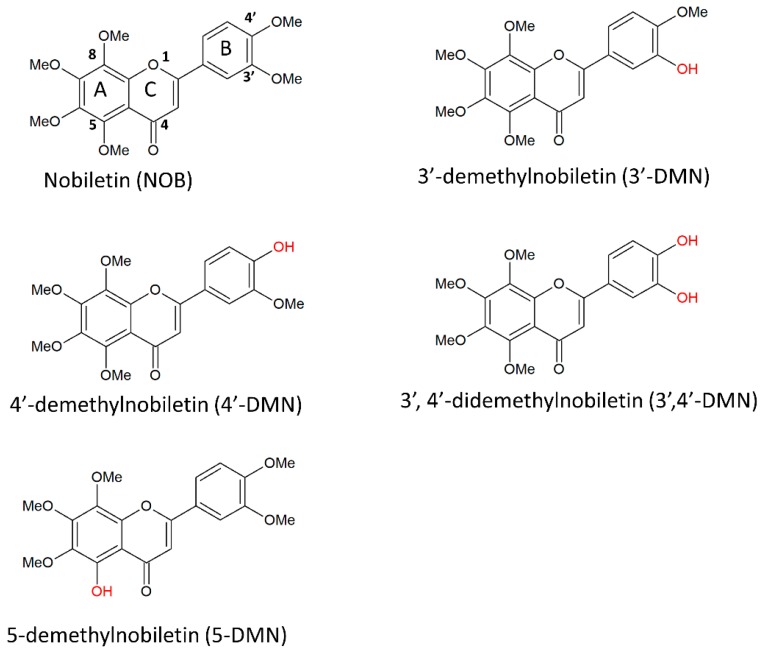
Chemical structures of nobiletin and its derivatives.

NOB is a PMF classified under the flavonoid family of polyphenols. The International Union of Pure and Applied Chemistry (IUPAC) nomenclature is 2-(3,4-dimethoxyphenyl)-5,6,7,8-tetramethoxychromen-4-one. It is also known as 5,6,7,8,3′,4′-hexamethoxyflavone or 2-(3,4-dimethoxyphenyl)-5,6,7,8-tetramethoxy-4H-1-benzopyran-4-one [12]. NOB has a molecular formula of C_21_H_22_O_8_ and a molecular weight of 402.399 g/mol. The chemical structure of NOB is illustrated in Figure 1. This flavone has a distinct three aromatic ring structure (labelled A, B and C in Figure 1), with the ketone and ether group in ring C along with four methoxy groups at the 5, 6, 7 and 8 positions of ring A and 2 methoxy groups at the 3 and 4 positions of ring B. Under long-term storage, NOB can degrade into 5-demethylnobiletin (5-DMN), IUPAC name 5-hydroxy- 6,7,8,3′,4′-pentamethoxyflavone (structure illustrated in Figure 1), through the process of autohydrolysis [49]. It has also been proposed that 5-DMN could be formed through conversion of nobiletin by gastric acid after oral consumption [50].

Both the NOB and 5-DMN undergo further transformation to form a number of metabolites in the body after ingestion [50,51]. More than 20 metabolites have been identified and the types vary significantly according to the species of citrus plants [12]. The three common phase I metabolites of NOB identified in urine after administration to rodents are 3′-DMN, 4′-DMN and 3′,4′-DMN [52,53]. Wu et al. successfully quantitated the amount of NOB, 3′-DMN, 4′-DMN and 3′,4′-DMN at 2.03, 3.28, 24.13 and 12.03 nmol/(gram of tissue of colonic mucosa) at the end of 20 weeks daily feeding of 500 ppm NOB to CD-1 mice [54].

After absorption, NOB generally undergoes Phase I and Phase II metabolism. In vivo tests show the Phase I demethylation of NOB is likely caused by the action of cytochrome P450 [55]. Koga et al. researched the enzymes involved in NOB metabolism and confirmed that CYP1A1, CYP1A2, CYP1B1 and CYP3A5 are involved in the conversion of NOB to 3′-DMN; further action from CYP1A1 and CYP1A2 is required to convert 3′-DMN to 3′,4′-DMN [56]. NOB was also found to undergo extensive Phase II metabolism in the small intestine involving glucuronides or sulphates. [57] Four phase II metabolites of NOB have been identified in rodent serum, bile and urine. These Phase II metabolites are formed from the glucuronidation/sulphation of the Phase I products, namely 4′-DMN and 3′,4′-DMN [58]. However, research on these Phase II metabolites are limited likely due to the fact that existing literature suggests a high likelihood that these substances have decreased activity. For example, Manthey et al. showed there was a reduced anti-inflammatory effect of the compound after glucuronidation [59].

In contrast to the dominant Phase II metabolites in the small intestines, the majority of the metabolites in the large intestine undergo deconjugation mainly through the action of the microflora in the gut. The microbiome produces enzymes such as C-deglycosidases, O-deglycosidases and hydrolases that break down the unabsorbed compounds from the small intestine. The microbiome also releases enzymes such as glucuronidases and sulphatases that hydrolyze the conjugate bonds, resulting in the reformation of free molecules that either undergo reuptake into the colonocytes or enter into the blood stream [60]. At present, only a limited species of the microbiome have been identified and further research is crucial to understand the in vivo biotransformation of the NOB compound resulting in the generation of multiple metabolites with different activities [61]. It is likely that the subtle variances in the gut microbiome in different individuals may result in different pharmacodynamic effects after administration of NOB. For instance, 4′-DMN and 3′,4′-DMN have been shown to exhibit higher anti-cancer and anti-inflammatory effects than NOB itself, but the rate of conversion from NOB to these metabolites may vary from one person to another [54,62]. The mechanisms of NOB in chemoprevention are elaborated under ‘Section 5—Chemopreventive effects of NOB, 5-DMN and NOB-metabolites’.

Early in vitro studies using rat liver S9 extracts reveals 3′-DMN as the main metabolite of NOB after 24 h of treatment [42]. However, further High-Performance Liquid Chromatography (HPLC) analysis on in vivo experiments showed that the concentration of nobiletin and its metabolites differ in the colonic mucosa—the concentration of 3′-DMN is almost equal to NOB, while 3′,4′-DMN is about 5.9-fold more than NOB, and 4′-DMN being the most concentrated, at 11.9 times the concentration of NOB. Integrating these values, the concentration of NOB is actually 20 times significantly lower in the colon when compared to the total concentration of its metabolites [54]. Convincing evidence has shown that these metabolites generated in vivo following oral administration of NOB result in significant accumulation in colonic tissues which is associated with the chemopreventive effect for CRC.

Interestingly, growing evidence suggests that the metabolites have more potent anti-cancer activity than their parent compounds, and the high concentration of the metabolites of NOB found in the colon may indicate that anti-cancer effect of NOB is largely conferred by its metabolites. This is consistent with the findings of Wu et al. who discovered that by treating HCT116 cell lines with NOB and its metabolites results in a 3.3 to 7.6-fold increase in apoptotic cells [54]. A recent study by Chiou et al. also shows that the hydroxylated PMF, 5-DMN is more potent than NOB in terms of its chemopreventive effect on colon malignancy for both in vivo studies using xenograft mice and in vivo studies using three different colon cell lines. Chiou and colleagues reported that 5-DMN shows different levels of inhibition in different types of cell lines, with the highest efficacies in COLO205 cell lines, followed by HCT116 and HT-29 [49]. This is consistent with the findings of Qiu et al. stating that the half maximal inhibitory concentration (IC_50_) required for 5-DMN to exert an inhibitory effect on the growth of HCT116 cells is 8.4 μM as compared to the notably higher value of 37 μM for NOB. Similarly, the IC_50_ required for 5-DMN against HT-29 cells is 22 μM as compared to the higher IC_50_ of 46.2 μM for NOB [63]. This may suggest that the hydroxyl group at the 5th position on the A ring is an important functional group involved in the molecular interactions [49].

## 4. Pathogenesis of Colorectal Cancer

The mechanisms leading to CRC development are part of a rather complex process. The pathogenesis of CRC is arbitrarily subdivided into three stages here: initiation, progression and metastasis. Each pathway is known to be regulated by chemical signals, called cytokines, which allow the progression from one stage to the next, whilst inflammation is the underlying result of each stage [11].

Cancer generally starts with a mutated cell which deviates from the normal cell growth cycle and progresses through the cell cycle rapidly with no differentiation of structure or function. It can be attributed to the down regulation of the regulatory genes or up-regulation of oncogenes. This gradually leads to the formation of a mass of undifferentiated cells called an adenoma. This lump of cells does not perform any specific function but competes with the surrounding normal cells for nutrients. In more than 60% of colorectal adenomas, the dysregulation of adenomatous polyposis coli gene resulting from the Wnt/β-catenin pathway is the major culprit in triggering this process [64].

The initial adenoma progresses on to an intermediate adenoma when the epidermal growth factor receptor (EGFR) is activated, which in turn triggers the phosphatidylinositol-3-kinase pathway and results in tumour formation [65]. Also, the inactivation of transforming growth factor-β (TGF-β) and the loss of function of p53 further aggravates tumour growth by preventing apoptosis [66,67].

Eventually, a tumour becomes malignant when angiogenesis occurs, and the cancer cells are released into the bloodstream and spread to other parts of the body through a process known as metastasis. Intercellular adhesion molecule-1 (ICAM-1) and matrix metalloproteinase (MMPs) are closely associated with the promotion of angiogenesis and metastasis. To illustrate, the MMP disrupts the integrity of the basal membrane allowing the cancer cells to enter the surrounding blood vessels and thus the blood stream through a process known as intravasation [68,69].

## 5. Chemopreventive Effects of Nobiletin, 5-DMN and NOB-Metabolites

In one of earliest in vitro studies, the antiproliferative effect of NOB was evaluated against HT-29 colon cancer cells [70]. The study determined that the IC_50_ and IC_90_ of NOB against HT-29 cell were 4.7 μM and 13.9 μM, respectively, via the 3H-thymidine uptake assay [70]. As a product of autohydrolysis of NOB, 5-DMN was also evaluated for its antiproliferative effect against colon cancer cells. In the H-thymidine uptake assay, the IC_50_ and IC_90_ of 5-DMN against HT-29 was reported to be 8.5 μM and 171 μM, respectively [70]. In the following years, NOB and 5-DMN were also reported to be cytotoxic towards different colon cancer cell lines, including HCT116, HT-29, SW489, COLO320, COLO205 and Caco-2 (Table 1). Despite the stronger anti-proliferative effect of NOB observed in the earlier study [70], recent studies increasingly showed that 5-DMN exhibits stronger cytotoxic effects against different colon cancer cells as compared to NOB [49,63]. These contradictory results are potentially due to the different aspects of cancer focused in each study. Based on these in vitro studies, NOB and 5-DMN were shown to exhibit their cytotoxic effects towards colon cancer cells, predominantly via cell cycle arrest and induction of apoptosis (Table 1).

Multiple in vivo studies demonstrated that NOB offers a protective effect against several carcinogens, such as the azoxymethane (AOM) and the 2-amino-1-methyl-6-phenylimidazo[4,5-b]pyridine (PhIP) (Table 2). AOM/DSS has been used to induce colitis in mice for the purpose of creating mouse models that replicate colitis induced CRC in humans [71]; however, PhIP, a heterocyclic amine, is a food-derived carcinogen that is abundantly released in the process of cooking fish and meat [72,73]. Administration of 0.01% wt of NOB to mice for five weeks in their diet resulted in the reduction of abnormal growths induced by colonic carcinogen AOM in the colons of the mice; there was a 50% reduction as compared to the controls [41]. Another similar study to determine the anti-adenocarcinoma effects of NOB also showed positive results but with lower efficacies, whereby 34 weeks administration of 0.01% or 0.05% wt. of NOB reduced the frequency of adenocarcinoma by 12% and 32%, respectively [74]. In addition to that, Wu et al. demonstrated that NOB treatment successfully reduced the rate of cell proliferation by 69%, tumour incidence by 40%, tumour multiplicity by 71%, and downregulated TNF-α, IL-1β and IL-6 by 65%, 69% and 45% respectively in AOM/DSS treated mice [54]. Consistent with the inhibitory effect against AOM induced colon carcinogenesis, NOB also showed significant reduction in the high density of colonic aberrant crypt foci (ACF) located in the transverse colon in PhlP-induced F344 rats [75]. This shows that NOB is effective in preventing CRC triggered by different types of carcinogens.

Further support for NOB as a prospective candidate for chemoprevention is that NOB is known to inhibit different pathways leading to cancer via a number of different mechanisms which includes inhibiting cell cycle progression [54,76], limiting inflammation [76], inducing apoptosis [54], preventing angiogenesis [77] and reducing tumour formation [49,54,78]. This subsection will describe the mechanism of action of NOB, its autohydrolysis product, 5-DMN and its three common metabolites, namely 3′-DMN, 4′-DMN and 3′,4′-DMN, in chemoprevention of CRC in detail.

### 5.1. Cell Cycle Arrest

Uncontrolled cell growth that arises from genomic instability is known to contribute to tumorigenesis [85]. One way to counteract CRC is to halt its cell cycle progression. The cell cycle is akin to a biological growth clock that tightly regulates each stage of cell growth, where any mutated or abnormal cells will be arrested at either the G1 or G2 checkpoints; however, this mechanism is disrupted in cancerous conditions [86]. To progress through the stages, the regulatory protein cyclin acts like a key, as it needs to phosphorylate the cyclin-dependent kinase (CDK) complexes to allow progression to the next stage [87].

#### 5.1.1. Action of NOB and Its Metabolites Inducing Cell Arrest

Notably, different metabolites of NOB work by different mechanisms against different cells. The flow cytometry test showed NOB and 4′-DMN arrest cells at G0/G1 phase in both HCT116 and HT-29 cell lines, despite the inhibitory effect of 4′-DMN being higher than that of NOB. Both 3′-DMN and 3′,4′-DMN arrest cells at both S phase and G2/M phase in HCT116 cell lines but arrest cells at both G0/G1 and G2/M phase in HT-29 cells. The inhibitory effect of 3′,4′-DMN is higher than that of 3′-DMN as only half the concentration is needed to induce a similar end result [54]. The fact that the metabolites 3′-DMN and 3′,4′-DMN exhibit more potent anti-cancer effects than NOB may suggest that demethylation at the 3′ and 4′-position significantly enhances its inhibitory effect [51].

In vitro tests using HCT116 cells reveal NOB and all three of the common metabolites increase the expression of CDK inhibitor, p21^Cip1/Waf1^ [54]. p21^Cip1/Waf1^, also known as p21 or P21/CDKN1A is a negative regulator for progression of the cell cycle that is responsible for the hypo-phosphorylation of retinoblastoma (Rb) proteins, leading to cell cycle arrest at the G1/S transition [86,88,89]. Although p21 is usually associated with the degradation of cyclin D1 [86], it is interesting to note that only 4′-DMN but not other metabolites nor the NOB itself causes significant reduction in cyclin D1 level. This may partly explain the strongest cell cycle arresting effect of 4′-DMN at the G0/G1 phase as compared to the other compounds aforementioned [54].

Proliferating cell nuclear antigen (PCNA) acts as a cofactor for DNA polymerase δ. It is an important marker commonly used to detect cell proliferation due to its increased expression through the G1 phase and S phase transition of cells [90,91]. Analysis from immunohistochemical tests recorded 69% reduction of cells with PCNA compared with the untreated controls [54]. Interestingly, evidence also reveals that p21 potentially suppresses action of PCNA. Interaction with the carboxy terminal of p21 inhibits PCNA from activating DNA polymerase δ, thus blocking DNA synthesis and preventing cell proliferation [92,93]. In this light, specific research of NOB on p21 and PCNA may be required to elucidate the pathways in further details.

Wu et al. studied the combinatory effect of NOB and its metabolites at different concentrations on HCT116 cells. At half the original concentration present in the colon, there is a decreasing trend of cells in S phase and G2/M phase but an increasing trend was noted in the G0/G1 phase. The cell cycle arrest effect seems to be dose dependent as flow cytometry recorded the population of cells arrested at the G0/G1 phase to be 57.8% higher than the untreated cells and significantly increased to 91.0% when the concentration of NOB and metabolites was doubled. To validate the findings, the levels of key signalling proteins were measured. Results showed that treatment with NOB and its metabolites lowered the levels of CDK-2, CDK-4, CDK-6 and cyclin D, raised the level of p52 and p27, but did not alter levels of p21 and cyclin E. In contrast, in vivo tests in AOM/DSS induced mice solely treated with NOB did document decreased expression of cyclin E and increased expression of CDK inhibitor p21. This difference is hypothesised to be due to the cell type specific response towards NOB, which is yet to be confirmed by further research [76]. The cell cycle is tightly regulated by key signalling proteins such as cyclins and cyclin dependent kinases (CDKs). To illustrate, complexes such as cyclin D-CDK-4/6 and cyclin E-CDK-2 facilitates the transition from G1 phase to S phase, while cell transition from G1 phase to S phase can be inhibited by tumour suppressor p53 and CDK inhibitors p21 and p27 [89,91].

It is also worth mentioning here that, at the effective concentration that arrests cell cycle, NOB produces a cytostatic effect, meaning it arrests growth without killing the cell [14]. As compared to other flavonoids like tangeretin (IC_50_ = 1.6 μM) and quercetin (IC_50_ = 0.84 μM), a slightly higher IC_50_ of 4.7 μM is required for the cell proliferation inhibition action by NOB in HT-29 cell lines and IC_50_ of 8.4 μM for 5-DMN [70]. However, the inhibitory effect of NOB may only be temporary. It is demonstrated that, with the removal of NOB, the treated cells resume cell proliferation within 24 h and regain similar growth status comparable to the control within 96 h [14]. This may also imply that, in order to sustain the inhibitory effect of NOB, the treatment with NOB has to be long-term to ensure continuous cell proliferation inhibition. This is possible as NOB is considered a natural compound and has no effect on healthy cells [83]. One might argue that the effect of NOB may be problematic for naturally fast-proliferating cells like healthy non-adenomatous intestinal lining cells. However, there is reassurance based on previous research that showed NOB is 10 times more selective towards transformed cancerous cells as compared to normal healthy cells [79].

#### 5.1.2. Action of 5-DMN Inducing Cell Cycle Arrest

Treatment with 5-DMN also shows a similar increase of Rb in a dose dependent manner. Notably, 5-DMN does not affect the level of CDK-4, but there is a significant reduction of CDK-2 levels [63], hence indicating a reduced possibility of complex formation with cyclin A or cyclin E [94]. p21 is known to play a key role in arresting the cells at the cells at the G2/M phase through the inhibition of CDK-2/Cyclin E complex formation [95,96], and 5-DMN has been found to be able to arrest cell cycles at both the G0/G1 phase and G2/M phase in HCT116 (p53 ^+/+^), but is only able to accumulate cells at the G2/M phase in HCT116 (p53 ^−/−^). This suggests that G0/G1 arrest is dependent on p53 while G2/M is independent of p53 [80]. Using HT-29 cell lines, Qiu et al. reported that 5-DMN effectively causes cell cycle arrest at the G2/M phase [63]. This effect possibly arises from the downregulation of cdc25 protein expression, which is important to activate the cyc2/cyclin B1 through the process of dephosphorylating the inactive tyrosine residues Thr-14 and Thr-15 located in cdc2 ATP binding domain [97,98].

To sum up, different derivatives of NOB potentially arrest the cell at different stages of the cell cycle, mainly through downregulating the expression of proteins or kinases such as CDKs involved in the cell proliferation pathways and preventing the formation of cyclin complexes that allow cell cycle progression.

### 5.2. Programmed Cell Death

As growth of a cell is tightly regulated by the cell cycle, death of a damaged or aged cell also needs to be programmed to maintain homeostasis in our body. There are three models of programmed cell death (PCD), namely apoptosis, autophagy and necrosis [99]. A tumour mass of cancerous cells is formed when the cancerous cells develop the ability to evade cell death. Not responding to the death signal, the cells continue to grow and proliferate, leading to progression of cancer. Thus, NOB, being an agent that targets the key signalling pathways of programmed cell death, may help in chemoprevention of CRC. Apoptosis will be the main focus in this subsection, while autophagy will only be discussed briefly. Necrosis, the most abrupt death of all three, will not be discussed in this section as there are no data in this area. Although necrotic death is usually associated with inflammation, this does not exclude its possibility to be exploited as a means to eliminate cancerous cells [100].

Apoptosis can be induced by two core mechanisms, namely the extrinsic and the intrinsic pathway. The cell death signals in the extrinsic pathway come from external sources such as the Fas ligands or tumour necrosis factors [101]. The intrinsic pathway generally arises from the mitochondrial intracellular protein of the Bcl-2 family. Bcl-2 is an important regulator for apoptosis, which plays a role in mitochondrial disruption that activates the caspases [102]. High levels of Bcl-2 are expressed in various types of cancer and is associated with chemoresistance. Levels of Bcl-2 need to be lowered to promote apoptosis [103,104]. As a result of reduced Bcl-2 levels, a cascade of activity is activated in the cell leading to apoptosis with caspase-9 acting as the initiator caspase in the intrinsic pathway [105] and caspase-8 in the extrinsic pathway. It may also be crucial to mention here that the procaspase-8 forms a complex called Death Inducing Signalling Complex (DISC) before it is activated to caspase-8. The downstream effect would be the activation of the executioner caspase-3, and other caspases such as caspase-1, caspase-6 and caspase-7 [106,107] which then cleaves Poly (ADP-ribosome) polymerase (PARP) [108,109]. Soon after, the cell starts to bleb and shrink while its nucleus is condensed and fragmentised, proteolysis happens and the cell loses adhesion to the extracellular matrix and neighbouring cells [110,111]. Once the cell undergoes apoptosis, its contents are taken up by the body and recycled for new cell synthesis.

#### Action of NOB and Metabolites Inducing Programmed Cell Death

In vitro tests using different colon cell lines such as HCT116 and HT-29 reveals that the action of NOB and its various metabolites vary in different cell types. NOB was only shown to induce apoptosis of colon cancer cells when tested at high concentration. Zheng et al. [79] demonstrated that NOB increased DNA fragmentation in COLO302 only at 200 μM. Treatment with NOB, 3′-DMN, 4′-DMN and 3′,4′-DMN in HCT116 cell lines raise the early apoptotic cell population by 3.3-fold, 5.0-fold, 4.9-fold and 7.6-fold, respectively, while also resulting in 4.2-fold, 3.5-fold 7.1-fold and 4.5-fold increments in the late apoptotic cell population, respectively. In contrast, 3′-DMN and 4′-DMN did not cause any significant changes in apoptotic cell population in HT-29 cell lines, but the pro-apoptotic effect of 3′,4′-DMN was observed to be higher than that of NOB. An in vitro test using HCT116 shows that all three metabolites of NOB are able to induce the activation of caspase-3, caspase-9 and PARP, while NOB can only induce activation of caspase-9 but not that of caspase-3 or PARP. A negative result was also reported on the apoptosis inducing effect of NOB, whereby no apoptosis was detected when NOB was tested at concentrations of up to 100 μM in HT-29 [14]. In contrast, an in vivo test in AOM/DSS treated mice revealed a 2.3-fold increase of caspase-3 levels with NOB treatment [54]. Nevertheless, we can be certain that the metabolites of NOB render a higher proapoptotic effect as compared to their parent compound NOB.

Several previous works have demonstrated that NOB and its derivatives exhibit pro-apoptosis properties. However, this effect is known to be tissue specific. To illustrate, apoptosis is observed in colon cell lines but not the HL-60, promyelocytic leukaemia cell lines [79,112]. Interestingly, apoptosis could also be related to MMP-7. It has been discovered that cells that express MMP-7 are less sensitive to the Fas ligand-induced apoptosis as MMP-7 has a higher tendency to produce the non-apoptotic form of soluble Fas ligand by releasing the ligands in the cell membranes [77]. MMP will be further discussed in Section 5.4.

In vivo tests using xenograft mice show that 5-DMN triggers apoptosis at a concentration of 40 μM [49]. 5-DMN has been reported to increase levels of caspase-8, caspase-3 and PARP in a dose dependent manner, and results in 2.2-fold increase in the population of early apoptotic cells as compared to the control. In contrast, there is no apparent increase in the apoptotic effect even after doubling the concentration of NOB, suggesting NOB is required at a significantly higher concentration to induce a pro-apoptotic effect [14,63]. However, the effect of 5-DMN was notably distinct for different types of cancer cell lines. To illustrate, 5-DMN can induce early apoptosis in HCT116 colon cancer cell lines at a concentration as low as 8 μM while 5-DMN only slightly raises the apoptotic activities at a concentration as high as 36 μM in colon HT-29 cancer cell lines [63].

Annexin-V/PI analysis reveals that 5-DMN significantly increased the Annexin-V positive cells, especially in the late apoptotic or necrotic cell population among the HCT116 (p53 ^−/−^) cells, suggesting that the action of 5-DMN may be independent of p53 [80]. The fact that 5-DMN induces early apoptosis in HCT116 (Bax ^+/+^) cells but not in HCT116 (Bax ^−/−^) may suggest that Bax (Bcl-2 associated X protein) is important for apoptosis to occur. In other words, absence of Bax confers resistance to apoptosis [80,113,114]. A recent study by Chiou et al. proved that 5-DMN increases the expression of p53 proteins, which not only induces apoptosis, but also triggers cell death by autophagy that contributes to the prevention of tumour growth [49]. However, the detailed mechanism of how NOB prevents CRC development through the autophagy process is yet to be elucidated. From the current stage of knowledge, autophagy is known to exhibit both the pro-tumour and anti-tumour formation effects. In response to stress, autophagy acts as a protective mechanism for cell survival, which has already been elaborated in previous literature [115]. It is likely that overactivation of autophagy contributes to suppressing tumour formation by inhibiting the anti-autophagic-related genes (ATGs) in oncogenesis and activating the pro-ATG [116]. The effect of autophagy and apoptosis may be synergistic in chemoprevention of cancer [117,118].

To conclude, NOB was shown to be less effective in inducing apoptosis of colon cancer cells. Instead, the metabolites of NOB, 3′-DMN, 4′-DMN and 3′,4′-DMN were suggested to be responsible for the induction of apoptosis of colon and modulation of cancer cell growth in the colon carcinogenesis animal model [54] On top of that, the autohydrolysis product of NOB, 5-DMN could induce apoptosis in colon cancer cells at a lower concentration as compared to NOB.

### 5.3. Anti-Inflammation

Inflammation is a natural physiological response of our body, characterised by five main signs, namely loss of function, redness, pain, heat and swelling. Inflammation plays a significant role at times of infection and injury. It triggers the immune system to function and helps to protect our body through the release of chemical molecules called pro-inflammatory signals. However, too much inflammation is also not a good sign. There is increasing evidence narrating the interrelation between tumorigenesis and inflammation [119]. Whilst chronic inflammation is a hallmark of cancer, the inflammatory cytokines aggravate cancer progression by preventing differentiation of cells and promoting tumour formation [120]. The inflammatory cells release ROS after being activated, leading to the oxidative damage of DNA and p53 mutation [121,122,123]. The mechanism that triggers inflammation is a rather complex pathway and has been covered by previous literature [123,124]; only the anti-inflammatory effects mediated by NOB are discussed in this review.

Increasing evidence shows that progression of CRC can be accelerated by the upregulation of pro-inflammatory cytokines expressions—for example, TNF-α, IL-1α, IL-1β and IL-6 [81,119,125]. These proinflammatory cytokines enhance the secretion of inflammatory mediator PGE_2_. Song et al. reported that treatment with NOB results in a noteworthy reduction of tumour size and frequency correlated with the significant lowering of IL-1, IL-6, iNOS and COX-2 levels [78]. ELISA test quantified the reduction of TNF-α, IL-1β and IL-6 at 51%, 92% and 69%, respectively, in the NOB treated group while real-time qRT-PCR quantified the reduction of the above pro-inflammatory cytokines at 65%, 69% and 45%, respectively, when compared to the control mice [54].

#### Anti-Inflammation Effect of NOB and Its Metabolites

Besides NOB, multiple studies have shown that its metabolites, especially, 4′-DMN and 3′,4′-DMN, also exhibit significant inhibitory effects towards nitric oxide production, iNOS and cyclooxygenase (COX) expressions in both in vivo and in vitro conditions [30,31,62,76,81,126]. However, the combined effect of NOB and its metabolites warrants further investigation [76]. Notably, NOB selectively inhibits COX-2 and did not affect COX-1 [81]. COX-2 is normally absent in healthy cells, but its release is triggered when the environment is inflammatory or hypoxic [127]. COX-2 is known to enhance CRC carcinogenesis, and inhibiting COX-2 also limits the production of PGE_2_ [128], which may be associated with the inhibition of cell proliferation in colonic mucosa [41]. iNOS speeds up the conversion of L-arginine to NO through the process of oxidative deamination. NO is a potent inflammatory mediator that activates signalling molecules that trigger the process of inflammation and mutagenesis [129]. By inhibiting the iNOS and its downstream products, NOB helps in reducing the inflammation observed in chronic diseases like ulcerative colitis and CRC [130]. Introduction of NOB to colonic tissues harvested from mice treated with AOM/DSS results in a 35% reduction of cells expressing iNOS compared to the untreated tissue. This is consistent with the in vitro test. By administering NOB and its metabolites at a concentration equivalent to that found in the colons to the LPS-induced RAW 246.7 macrophages is shown to effectively and completely inhibits expression of iNOS, while, at half the concentration, the expression of iNOS was lowered by 56.4% compared to the untreated LPS-induced macrophages [76]. This shows that a similar process is likely to happen in the human body and NOB is indeed a promising anti-inflammatory agent.

Additionally, NOB also increases the release of the Nrf2-dependent enzymes which regulate Phase II enzyme production, such as heme oxygenase-1 (HO-1) and NQO1. HO-1 is an anti-oxidative enzyme that exhibits its anti-inflammatory effect by producing anti-oxidants like carbon monoxide and bilirubin. It is also important to note that HO-1 is not solely controlled by Nrf-2 [76]. NQO1 upregulation counteracts the increased expression of IL-1β and TNF-α induced by LPS [131]. This is consistent with the findings of Khor et al. reporting that a lower Nrf-2 expression greatly increases susceptibility of mice models to AOM-induced colitis [132]. The colonic mucosa of AOM/DSS-induced mice orally administrated with NOB was found to have increment of nuclear Nrf2 by 1.94-fold and reduction of cytoplasmic Nrf2 by 36% when compared to the AOM/DSS-treated mice. The upregulation and translocation of Nrf2 transcription factor is thought to be the cause for subsequent increment of HO-1 and NQO1 by 2.78-fold and 2.59-fold, respectively. This is consistent with the results from the combinatory effect of NOB and metabolites treatment on macrophages cell lines, which induces a 10% increase in the level of HO-1 and a 34% increase in the level of NQO1 when the concentration ratio of NOB and metabolites is equivalent to that in the colon [76]. In short, Nrf-2, which neutralises carcinogens and reactive oxygen species (ROS), is identified as a key signalling pathway to target in the effort to halt CRC progression [133,134].

### 5.4. Anti-Angiogenesis

Angiogenesis refers to the process by which new blood vessels are formed. This process is an important pathway that results in the progression of all types of cancer. This is because, when the tumour mass grows, it naturally needs more nutrients and nourishment to support its growth. To achieve this necessity, new blood vessels have to be formed surrounding the tumour mass to ensure a continuous supply of oxygen and glucose to support the growing cell mass [135,136].

#### Anti-Angiogenesis Effect of NOB

It is postulated that NOB prevents metastasis by inhibiting the activity of activator protein-1 (AP-1), a dimeric protein, thus preventing DNA binding [77]. Another hypothesis suggests that NOB acts via the Nuclear Factor-kappa B (NF-κB) pathway, altering the gene expression by modulating the promoter regions [126,137].

For angiogenesis to occur, the vascular endothelial growth factor (VEGF) plays a key role. VEGF not only acts as a signal to induce new blood vessel growth, but also inhibits the apoptosis induction. VEGF works by activating the mitogen activated protein kinase (MAPK). This kinase triggers the signal transduction and allows the endothelial cells to proliferate in order to form new blood vessels [138,139,140]. To elaborate on VEGF, it is necessary to mention leptin and insulin-like growth factor 1 (IGF-1) here. There is evidence suggesting that bidirectional cross talk exists between the leptin protein and IGF-1, a serum growth factor. Acting together, they not only catalyse the cell proliferation process, but also transactivate the epidermal growth factor receptor (EGFR), which enhances the migration and invasion power of the cancer malignancy [141].

Miyamoto et al. discovered that leptin, a protein that regulates energy balance and body mass has a positive correlation with CRC, where leptin is thought to be a mitogenic factor that leads to the development of colon cancer [84]. It induces cell proliferation by activating the nuclear factor κB, p38 MAPK and p42/44 MAPK [142]. Previous evidence demonstrates that introduction of 0.1 to 10 nM of leptin enhances the proliferation rate of HT-29 cells by 1.3 to 1.6 times [84] through c-Jun NH_2_ terminal kinase and extracellular regulated kinase (ERK) 1/2 activation [143,144]. In other words, the chances of developing CRC can be reduced if leptin concentration is regulated. Treatment with NOB suppresses cell proliferation induced by leptin through inhibition of mitogen-activated protein/extracellular signal-regulated kinase (MEK) 1/2 [145]. Consistent with the in vitro findings, a reduction of 75% of leptin concentration by NOB, partly through the inactivation of the insulin signalling pathway, was reported at the end of the 17-week study in an in vivo model using Institute for Cancer Research (ICR) mice [83,84]. In this light, Miyamoto et al. conducted a study aiming to determine the prognosis of cancer in obese rats with flavonoid intervention. They reported that the flavonoids significantly reduced the incidence of β-catenin accumulated crypt (BCAC) by 64% to 71% and aberrant crypt foci (ACF) by 68% to 91% and proposed that this arises from the effect of NOB in downregulating the secretion of IGF-1 [83].

Metalloproteinase (MMP) plays a fundamental role in angiogenesis. MMP induces the protein that breaks down the extracellular matrix (ECM), thus making the blood vessel more permeable and allowing the cancerous cells to detach from the lump to flow, extravasate or invade the other parts of the body, causing the spread of tumour to other vital organs. This is a process called metastasis, leading to malignancy and cancer aggravation. The mortality rate greatly increases when cancerous cells metastasise to vital organs like the liver [146]. Similar to how a dexamethasone steroid acts, NOB is proven to be able to increase the expression of tissue inhibitor metalloprotease-1 (TIMP-1) in human synovial cells [81]. However, the benefit of upregulating TIMP-1 in CRC is debatable due to its bilateral role in cancer progression. Although TIMP-1 upregulation contributes to the anti-oncogenic effect, enhanced expression of it may lead to early phase tumour development via the pathways independent of MMPs. With this understanding, TIMP-1 glycosylation can function as a biomarker to aid in CRC staging [147].

There are more than 20 types of MMP involved in metastasis [148], with each one of them playing a distinct role [149]. However, whether MMP is produced by cancer cells or their surrounding stromal cells is still an ongoing debate [150]. Abnormally high levels of MMP-1, MMP-2, MMP-3, MMP-7, MMP-9 and MMP-13 have been implicated in CRC [150]. Treatment with NOB significantly inhibits release of pro-MMPs especially pro-MMP-7 (also known as metrilysin) mRNA in HT-29 cell lines. To illustrate, NOB at a concentration range of 25 μM to 100 μM, the proMMP-7 levels in the media diminishes significantly by 35% to 47% [77]. The maximal expression of MMP-7 arises from the β-catenin/TCF complex transcription factors formed in the presence of mutated APC genes [150,151,152]. Apart from MMP-7, the action of NOB on other MMPs in CRC is yet to be investigated. MMP-9, which is mainly secreted by inflammatory cells, is correlated with the transition phase from adenoma to adenocarcinoma, while the upregulation of MMP-3 usually suggests poor prognosis as it has a positive correlation with low microsatellite stability. On the other hand, high levels of MMP-12 reduces CRC mortality as it can potentially inhibit angiogenesis [150] by secreting angiostatin, a chemical that halts tumour progression and inhibits tumour neovascularisation [153,154,155]. As mentioned in the previous section, NOB suppresses MEK. This suppression of MEK then further diminishes the expression of pro-MMPs, which results in the reduction of MMP and subsequently confers anti-angiogenesis effect [144,145]. Briefly, NOB prevents angiogenesis and metastasis in CRC mainly via the inhibition of MMP, EGFR and VEGF through the regulation of leptin and IGF-1.

## 6. Pharmacokinetics, Bioavailability and Delivery Systems of NOB

The pharmacokinetic properties of NOB represent a key factor to be considered in an attempt to formulate it into a therapeutic product. Understanding the interactions between the compound and our body opens ways to creative strategies in solving the problem which require novel formulation in delivering NOB for chemoprevention purpose. For oral delivery, an important consideration is the bioavailability of the active compound. However, the bioavailability studies on NOB are limited [156]. Therefore, understanding the pharmacokinetic profile of NOB becomes even more crucial to assisting in the prediction of bioefficacy as the absorption, metabolism and elimination pattern indirectly affect its bioavailability.

There are many factors that affect the absorption of a compound; one important consideration is the molecular structure [157]. The proper absorption of any compound is depicted by its solubility and permeability across physiological barriers of which both properties are directly related to its molecular structure. Attributed to its unique chemical structure with multiple methoxy groups, NOB is lipophilic in nature and can easily pass through the cell membrane. Murakami et al. successfully demonstrated the relatively high permeation of NOB across differentiated Caco-2 cells which mimics the epithelial cells lining the small intestine. A significantly high 48.1% of NOB has been found to permeate through the basolateral compartment while another 39.3% remains on the apical compartment four hours after introduction of NOB in a Caco-2 monolayer trans-well permeability assay [158]. Parallel artificial membrane permeation assay (PAMPA) deciphered the permeability of NOB, 4′-DMN and 3′-DMN at 1.38 × 10^−6^ cm/s, 1.14 × 10^−6^ cm/s and 1.05 × 10^−6^ cm/s, respectively [159]. It was discovered that the methoxylated flavonoids show five to eight-fold higher permeability in the intestinal wall than its unmethoxylated counterparts [160]. The drawback is that PMF in general has limited solubility. Results from the high-throughput lyophilisation solubility assay (LYSA) reveal that NOB has a low solubility at 12 μg/mL, while its metabolites, 4′-DMN, 3′-DMN and 5-DMN exhibited two to three-fold higher solubilities of 22 μg/mL, 29 μg/mL and 32 μg/mL, respectively [156,161]. In general, the solubility increased with the number of hydroxyl group of the compound. This may also partly explain the higher activity of the derivatives of NOB as compared to its parent compound, which has a higher number of methoxy groups.

After absorption, NOB is found to be widely distributed throughout the body, as a significant amount of NOB could be detected in organs such as the stomach, small intestine, large intestine, brain, liver and kidney within four hours of single dose administration [162,163]. Interestingly, NOB was suggested to be absorbed through the muscularis layer of the gastrointestinal tract, especially the stomach tissue into the blood circulation given the distinctly higher concentration of NOB in the muscularis (390 ± 120 nmol/g) as compared to other organs [162]. Furthermore, it is also noteworthy that NOB was found to be distributed in the mucous membrane and muscularis from the large intestine (cecum, colon and rectum) of a rat at 4.3 ± 1.6 nmol/g after one hour of oral administration by gastric intubation [162]. More recent evidence demonstrated that the levels of NOB in the colonic mucosa of mice were 2.03 nmol/g of tissue after long-term oral administration of NOB (0.05 wt%) containing diet. Furthermore, Wu et al. [164] also suggested that the dose of NOB used (0.05 wt%) could be equivalent to approximately 100 mg/day for human oral consumption, which is achievable in humans.

After oral administration of NOB to rats, the mean plasma concentration of NOB was quantified in several pharmacokinetic studies. Wang et al. [163] reported that the plasma levels of total NOB and its metabolites could reach as high as 10 μg/mL (25 μM). Using a highly sensitive Liquid Chromatography-Mass Spectrometry/Mass Spectrometry-Electrospray ionisation (LC-MS/MS-ESI) method, the maximum concentration (C_max_) of NOB in rat plasma was determined at 0.4 μg/mL (1 μM) after oral administration of 5 mg/kg NOB [165]. Meanwhile, a maximum concentration of 1.78 μg/mL (4.4 μM) was measured by a validated HPLC method in rat plasma after oral administration of 50 mg/kg NOB [166]. In addition, another study by Manthey et al. [167] reported that a peak of NOB serum level of 9.03 μg/mL (22.4 μM) was detected by HPLC-ESI-MS in rats after oral gavage of 50 mg/kg NOB. Nevertheless, these studies demonstrated a relatively early peak time (T_max_) of 0.25 to one hour after oral administration of NOB in rats. This high rate of cellular uptake may be attributed to the highly hydrophobic nature of the compound rendered by the presence of six methoxy groups [162].

NOB undergoes extensive metabolism after being taken orally. As detailed in Section 3, NOB undergoes Phase I and Phase II metabolism after being absorbed in the small intestine where it may be conjugated to sulphate and glucuronide, and then again deconjugated by the microflora in the colon [60]. The three common phase I metabolites of NOB have been identified as 3′-DMN, 4′-DMN and 3′,4′-DMN [52,53]. Wang et al. found evidence of transformation of 3′-DMN and 4′-DMN into 3′,4′-DMN in the colon [163]. The liver is another important organ involved in metabolising NOB. Koga et al. identified three metabolites, demethylated at the 4, 6 or 7 positions respectively under the action of human liver microsomes when incubated aerobically with NADPH [56]. The metabolites exhibit distinct activity and distribution pattern in different areas of the body. It was found that 4’-DMN is the major metabolite present in the small intestine and liver while 3′,4′-DMN was predominantly present in the colon and spleen [163]. An in vitro test on NOB using rat liver S-9 extract shows that only 7% of NOB metabolites were detected towards the end of a 24 hour treatment, while 72.6% of NOB remains unchanged towards the end of the experiment [158]. This may be attributed to the slow rate of demethylation of NOB showed by Murakami et al. [162].

The elimination half-life of NOB from the blood plasma of a rat was reported as 1.8 h via a validated HPLC test [166] while Kumar et al. reported a terminal half-life of NOB at 4.75 ± 0.57 h following oral administration and a terminal half-life of 1.51 ± 0.61 h following parenteral administration using the LC-MS/MS-ESI method [165]. Despite the wide distribution throughout the body, concentration of NOB quickly diminishes with time and becomes undetectable in the serum, stomach, intestines, liver and kidney. Aside from the parent compound, mono-demethylated metabolites and conjugated NOB are detected in the urine, with the concentration of conjugated NOB revealing a time-dependent increment over a period of 24 h [162]. Since NOB is rapidly eliminated from the body, significant adverse effects reported after administration of NOB are rare.

Although the in vitro results were promising, most of the reported concentrations of NOB evaluated (>20 μM) were not achievable in physiological conditions as demonstrated by in vivo pharmacokinetic studies of NOB. Comparing the high experimental levels used against the relatively low peak plasma concentration—a mere 1.78 μg/mL (4.4 μM)—after one hour of oral administration of 50 mg/kg NOB [166] and the rapid elimination from the body [162] points out a limitation to utilizing NOB in its unaltered natural form as a clinical drug. In fact, the levels of NOB detected in the colonic mucosa ranged between 2 to 4 μM using the assumption that one gram of tissue is equivalent of 1 mL of volume [54,162]. However, there was a study demonstrating that NOB at lower concentration (≤5 μM) exhibited antiproliferative effects against colon cancer cells [70], perhaps indicating true promise for clinical use after all.

To further substantiate the notion of NOB for colon cancer chemoprevention, multiple in vivo studies have demonstrated that dietary treatment with NOB could inhibit colon carcinogenesis in rats [54,76]. As mentioned earlier, this may be related to the fact that, while bioavailability of NOB itself is low, much of its anti-CRC effect may be via its metabolites. Wu et al. [54] indicated that the NOB level in the colonic mucosa only accounted for <5% of the total levels of NOB and its metabolites after oral administration of NOB. The study further suggested that the NOB metabolites, which were formed as a result of phase I and II metabolism and biotransformation by gut microbiome, play an important role in colon carcinogenesis inhibition [54]. Although there is some suggestion that lower doses can have an effect on cancer, clearly, enhancement of NOB bioavailability is necessary and also represents a major challenge that needs to be addressed to achieve the desired therapeutic effect.

Given the importance of actually delivering adequate amounts of NOB to the target site to achieve chemopreventive activity, we also reviewed the delivery systems aiming to enhance the bioavailability of NOB in the gut. For chemoprevention of CRC, oral delivery represents the preferred route. There is a growing interest to formulate lipophilic natural compounds such as NOB into emulsion, as these systems not only improve the bioavailability of the active compound, but also reduce the rate of degradation during storage [168]. Yang et al. attempted to enhance the solubility of NOB by encapsulating NOB with citrus oil-based emulsion. The team discovered that dissolving NOB at a higher temperature and in an oil with log P close to NOB, such as bergamot oil, helps to increase solubility of the compound [169]. Yao et al. also experimented with the possibility of using self-microemulsifying drug delivery systems (SMEDDS) to improve the permeability of NOB in the rat intestines and reported that SMEDDS resulted in similar efficacies to micelles, but showed better absorption profile when compared to sub-microemulsions [170]. Self-assembled NOB proliposomes were also reported to improve the absorptive rate and confer longer mean residence time as compared to NOB suspension in rats [171].

Furthermore, Chen and colleagues demonstrated that, through the addition of hydroxypropyl methylcellulose (HPMC), the retention of NOB in nanoemulsion is increased by 25% [172]. Even though the fabrication of supersaturating nanoemulsion with the addition of HPMC aimed to improve the physical stability of NOB and prevent precipitation of NOB in the emulsion, the fabrication did not perform as expected at high NOB concentration where precipitation still occurred during storage and digestion process in the gut [172]. To address the issue of component precipitation in the emulsion system, a recent intervention of nanoemulsion-filled hydrogel matrix has been developed to stabilize NOB and prevent precipitation during delivery along the GI tract [173]. Interestingly, the hydrogels could provide a controlled release of NOB along the GI tract, thereby the hydrogel shrank at acidic condition pH 1.2 but swelled and burst at pH 7.4. Due to the lower bioaccessibility of NOB in hydrogel as compared to nanoemulsion during digestion, the nanoemulsion-filled hydrogel matrix could confer a sustainable absorption of NOB through a controlled release in the intestinal tract [173].

Aside from the liquid formulations, Onoue and colleagues proposed a solid formulation of NOB with the intention to further enhance the bioavailability in addition to solving the stability issues which showed a remarkable 13-fold increment in bioavailability compared to the nanosized NOB amorphous solid dispersion [174]. However, the results only quantitate the brain permeability, but the data for colon effect is still lacking. Further research is needed to establish the practicability and feasibility of each delivery method to address the bioavailability challenges before NOB can be used in aiding patients at high risk of CRC.

## 7. Toxicity

Although NOB is derived from a natural source, excessive intake of any substance might lead to some changes in the body. To illustrate, there are several case studies reporting that ingestion of products containing bitter orange causes adverse effects such as tachycardia and ventricular fibrillation [175]. To address this concern, a number of studies have been carried out to further evaluate this problem.

Body weight is commonly used as a surrogate marker for toxicity, whereby the weight is expected to reduce significantly if toxicity occurs. Wu et al. concluded there was no significant change in body weight, weight of liver, spleen appearance and behaviour throughout the length of their research, suggesting that the oral intake of NOB at the effective concentration 40 μM or 0.05% for a period of 3 to 20 weeks does not lead to adverse side effects [41,49,54]. This is consistent with the findings of Murakami et al. suggesting that NOB leads to no cytotoxic effects [42]. The action of NOB is more likely of a cytostatic nature rather than cytocidal as, at the concentration that inhibits cell proliferation, it does not trigger apoptosis. Furthermore, the treated cells continue to grow normally once the effect of NOB diminishes [14]. This shows that NOB could be a safer option for CRC treatment as it is less cytotoxic as compared to the available chemotherapy agents.

## 8. Commercial Uses

A search on Google Scholar using the keywords “nobiletin patents” gives about 1160 relevant results. This initial search was refined through the advanced search function for patents using the SciFinder database which helps to narrow down the number of patents directly and indirectly related to NOB to 300.

After a close analysis of the patents, we found that, among the 300 patents related to the concept of NOB, the largest portion of the total patents involves the usage of NOB in the medical, pharmaceutical and nutraceutical fields. The patents include both the application in traditional treatments and also western medications, where there are about 20 patents related to cardiovascular diseases [176,177,178], hypertension and hypercholesterolemia [179], roughly 10 patents targeting the central nervous system [180,181] or neurodegenerative disorders [182], diabetes [183,184] and obesity [185] each, and about five patents concerning body metabolism and hormonal functions, bone-related disorders [186], oral issues such as ulcer and halitosis [187,188], liver-related problems like hepatitis [189] and anti-infectives such as anti-bacterial, anti-viral [190] and vaccines, respectively. The patents also include a small number of NOB usage in diseases like prostate disease, asthma [191,192], allergy [193], eye relief, prevention and improvements of conditions like hair fall [194], dysuria [195] and muscular atrophy [196].

A large proportion of the patents are related to anti-cancer treatments, which account for almost 13% of the total patents. The types of cancers covered are broad, ranging from the more prevalent ones like lung cancer [197] and breast cancer [198] to those lower down the prevalence indices like uterine, liver cancer, oral [199], and skin cancer [19]. Application wise, some major areas that involve the usage of NOB compounds include the synergistic effect of NOB with existing chemotherapeutic agents targeting the multidrug resistance cancer [200] which aim to increase therapeutic efficacy as well as aiming to address the side effects from conventional chemotherapeutic treatments, especially diarrhoea [201]. Some common cancer inhibition pathways leading to cancer that are targeted by the compound include anti-angiogenesis [202], anti-proliferation and anti-tumour or anti-neoplastic effects.

The usage of NOB in nutraceutical industries is also extensive; there are up to 20 patents of beverages that contain NOB, with some of the fortified drinks claiming to have pharmacological effects [203]. Next in the line, comprising one-fifth of the total patents, are the methods of extraction [204,205,206], purification [207,208], preparation [209,210], manufacturing [44,211,212] analysis [213], drug delivery and pharmacokinetics information such as ways to improve absorption, solubility and bioavailability.

Apart from that, the use of NOB in fields other than medicine is also very broad, which includes about 20 patents in cosmetics [214,215,216], another 20 patents in the food industry, which include its use as preservatives [217], flavourings or food additives [218], and four patents in the agricultural industry, of which mostly it is used as pesticide controls. Last but not least, there are also patents of NOB usage in stem cell technology and genetic analysis.

## 9. Future Directions

While NOB and its metabolites seem to have tremendous potential as chemopreventive agents, at this juncture of time, more intensive research is needed to resolve the challenges that arise from the limitations of this compound. As mentioned earlier, NOB showed dose dependent anti-cancer effects, but the challenge is to increase its bioavailability to enhance the chemopreventive effect. This is important as oral administration seems to be a more promising route of administration at the moment as intraperitoneal injection has been associated with severe side effects such as ischemic stroke [36]. In addition, given that the chemopreventive metabolites appear to be formed by via metabolism within the gut, the oral route seems to be a promising way of delivering drugs to the target site.

Although several effective delivery systems were developed to enhance the bioavailability of NOB, studies on targeted-delivery of NOB to the colon are still limited. Despite having delivery systems that enhance aqueous solubility and bioavailability, a colon-specific drug delivery system is highly desirable for efficient drug delivery of NOB to the colon or where the colorectal cancer reside. In 2012, a folate-modified self-microemulsifying drug delivery system (FSMEDDS) was developed with the aim to improve solubility of curcumin and specifically target colorectal cancer cells mediated by the binding of folate receptors in facilitating the endocytosis of the formulation [219]. Given the previous evidence of the successful preparation of NOB in SMEDDS [170], this intervention could be possible to be implemented to facilitate specific uptake of NOB into colorectal cancer cells via the FSMEDDS and further coated by Eudragit® S 100 (Evonik Industries AG, Essen, Germany), which prevent dissolution of the formulation under the condition of pH < 7.5 [219,220]. Recently, a dual stimuli-responsive Pickering emulsion (pH and magnetic- responsive) reported may hold immense potential for the biomedical field, particularly in the treatment of colorectal cancer [221,222]; to achieve an active targeting of specific sites, an external magnetic field could be utilized to direct the movement and accumulation of the drug carrier at the targeted sites to exert their therapeutic effects [223].

In addition to that, biotechnology can be applied to biosynthetically produce NOB in larger yields as citrus peel may only contain a limited amount of this bioactive product. In this regard, Itoh et al. successfully isolated five genes from *C. depressa* which encode the flavonoids by *O*-methyltransferases (FOMT), a precursor for a number of flavonoids. Quercetin has been synthesised via this method and it is highly likely that the same enzyme is also involved in the biosynthesis of NOB, suggesting that it may also be possible for NOB to be synthesised using this strategy [39]. However, the data on the effectiveness in this application is still lacking as there is limited research that uses this method to synthesise NOB. Apart from biotechnology, the introduction of reliable, efficient and economical validation methods such as ultraperformance liquid chromatography coupled with quadrupole time-of-flight mass spectrometry (UPLC-Q-TOP-MS) which allows high rate of separation of PMF compounds within 12 min also opens up more possibilities for NOB to be marketed [224].

In addition, another major challenge of chemotherapy that we are facing today is the development of drug resistance in cancer treatment. One possible cause that results in chemoresistance may be attributed to the cancer stem cell (CSC). CSCs are known to play a crucial role in tumour formation as they possess unique characteristics including unlimited cell renewal capacity and the ability to evade drug penetration [225,226]. Seeing the limitation of the single cell in vitro model [227], Silva et al. came up with a brilliant method of culturing cells into a three-dimensional block, which they named a 3D spheroid. At day seven, the 3D spheroids mimic the tumour lump, with the undifferentiated cells in the outer region surrounding the hypoxic inner core. Experiments showed that 2.9-fold higher concentration is needed to exhibit the same effect reported in the two-dimensional cell model [47].

Interestingly, the concomitant exposure of NOB and its metabolites gives rise to synergistic effects that are distinct from the response caused by NOB alone [228,229]. Therefore, the combinatory effect of NOB and its various metabolites should be explored in order to establish a solid foundation of understanding of the synergistic effect of NOB and its natural metabolites generated through the biotransformation process. Apart from that, compelling evidence showed that NOB produces a synergistic effect in tumour growth inhibition when co-administered with atorvastatin. When used together, only half the minimal effective concentration of each drug is required to achieve the targeted therapeutic outcome. Wu and co-authors reported a series of mechanisms by which this combination works, namely through altering important cellular signals that triggers inflammation, inhibiting cell cycle progression, inducing apoptosis and preventing angiogenesis and metastasis [164,230]. In this light, the drugs already in the market can be combined with NOB and tested for their synergistic effects in inhibiting CRC. In addition, the combinatory effect of NOB and its metabolites needs to be further elucidated to achieve a precisely targeted biological action in CRC chemoprevention. More clinical trials in human subjects with due ethical considerations are warranted as disparity will certainly exist if the data is solely extracted from in vitro or animal tests.

## 10. Conclusions

While there is a significant research focus on cancer, science is still at an early stage in understanding this noxious condition affecting people from every segment of society, but answers are critical as cancer’s prevalence and variance are continuously on the rise. The current clinical practice in cancer treatment, which largely consists of the three broad fields, namely surgery, chemotherapy and radiotherapy, may be helpful to patients to some extent but more intensive and in-depth ongoing studies are needed in the quest for a panacea for cancer given the high mortality rates of this malady. Many more patients will be relieved from pain and suffering if scientific research can shine a light on the root causes of cancer and focus on its prevention so as to nip the problem in the bud before the need to treat it arises.

The advancement in science has allowed the discovery of numerous beneficial compounds offered by nature. It is reassuring to learn that NOB, a compound that is extracted from the ubiquitous citrus species confers a wide range of beneficial biological effects that includes cancer prevention. On top of that, the autohydrolysis product, 5-DMN and several metabolites of NOB such as 3′-DMN, 4′-DMN and 3′,4′-DMN, demonstrate more potent effects as compared to their parent compound NOB. It is apparent that NOB is indeed a prospective compound that exhibits a promising chemopreventive effect on CRC, especially for the types which are induced by carcinogens or associated with diseases such as colitis. In addition to that, this review also focuses on the underlying molecular mechanism of which NOB acts in CRC. The plus point is that NOB and its products target a number of different hallmarks of cancer. To illustrate, NOB is endowed with anti-proliferative, pro-apoptotic, anti-inflammatory and anti-angiogenesis effects, which renders it the potential to counteract the pathology of CRC in patients at various stages of cancer progression.

Besides NOB, many compounds under the polymethoxyflavones family are currently promising candidates in the field of cancer research, yet it is too early for science to conclude a best compound to formulate as the elixir. More studies, be it in vitro, in vivo or clinical studies, are needed to unravel the full potential of each possible compound. Furthermore, it would be worthwhile to explore the synergistic effect or possible interactions between NOB and well-known anti-cancer drugs by both experimental and clinical studies. The vast number of existing patents of NOB across various industries may suggest that this compound does have commercial value besides its noteworthy pharmacological benefits. Further research work needs to be intensified to overcome the current gap and limitation in formulation, for instance to increase the bioavailability and to enhance the efficacies of NOB in CRC chemoprevention. Although significant advances have been made, there is still a long way to go before NOB could truly become part of the arsenal of CRC chemoprevention.

## Figures and Tables

**Table 1:** In vitro chemopreventive properties of NOB, 5-DMN and NOB-metabolites.

Compounds	Activities	Cell lines	Treatment/Assay (Treatment Duration)	Assays/Results/Mechanisms	References
NOB	Anti-proliferative	HT-29	H-thymidine uptake assay	- IC_50_ of NOB = 4.7 μM	[70]
				- IC_90_ of NOB = 13.9 μM	
5-DMN				- IC_50_ of 5-DMN = 8.5 μM	
				- IC_90_ of 5-DMN = 171 μM	
NOB	Cytotoxicity	COLO320, SW480 and Caco-2	MTS viability assay (48 h)	- IC_50_ for COLO320 = 40.4 ± 9.1 μM	[79]
				- IC_50_ for SW480 = 245 ± 9.1 μΜ	
				- IC_50_ for Caco-2 = 305.6 ± 41.9 μΜ	
	Apoptosis-inducing		Apoptosis assays—DNA fragmentation	- DNA ladder pattern	
				200 μΜ—2-fold increase DNA fragmentation in COLO320	
			- gel electrophoresis (48 h)		
	Anti-proliferative		BrdU labelling index	- 34.7 ± 4.7% BrdU-binding cells at 100 μΜ	
				- 44.4 ± 6.4% BrdU-binding cells at 40 μΜ	
NOB	Anti-metastasis	HT-29	ELISA		[77]
			- proMMP-7 expression	- At 100 μM, no detection of proMMP-7 in media, ~280 pg/mL proMMP-7 in media	
			qPCR and Western blot	- >25 μM, reduced RNA and protein (both intracellular and supernatant) expression of proMMP-7	
			AP-1 binding activity	- Inhibited binding activity of AP-1 (transcription factor for MMP-7 gene)	
NOB	Anti-proliferative	HT-29	Cell counting assay	- IC_50_ of NOB ≈ 50 μM	[14]
				- Inhibited cell proliferation in a time- and dose-dependent manner	
	Cell cycle arrest				
			Cell cycle analysis	- Induced G1 phase cell cycle arrest (60 and 200 μM)	
			- Propidium iodide staining		
	Apoptosis-inducing		Apoptosis assay	- No significant apoptosis detected at 60 and 100 μM	
			Resumption of proliferation	- Resumed proliferation within 24 h of removal of NOB and achieve the same stage of growth as compared to control after four days of removal of NOB	
NOB 5-DMN	Cytotoxicity	HCT116, HT-29	MTT viability assay (48 h)	- IC_50_ of NOB on HCT116 = 37 μM	[63]
				- IC_50_ of 5-DMN on HCT116 = 8.7 μM	
				- IC_50_ of NOB on HT-29 = 46.2 μM	
				- IC_50_ of 5-DMN on HT-29 = 22 μM	
	Cell cycle arrest		Cell cycle analysis - Propidium iodide staining (24 h) Western blot	- At 8 μM, 5-DMN induced G2/M phase arrest in HCT116	
				- At 36 μM, 5-DMN induced G2/M phase arrest in HT-29	
				- At 16 μM, NOB reduced CDK-2 expression	
				- At 4 μM and 8 μM, 5-DMN increased p21 and Rb, while decreased CDK-2 and p-Rb.	
	Apoptosis-inducing		Apoptosis assay	- At 8 μM, 5-DMN increased early apoptosis by 2.2-fold in HCT116	
			Annexin-V/PI (48 h)	- At 36 μM, 5-DMN increased early apoptosis by ~2-fold in HT-29	
			Western blot	- At 16 μM, NOB did not increase apoptotic cell population in HCT116/HT-29	
				- At 4 μM and 8 μM, 5-DMN increased expressions of cleaved caspase 8, cleaved caspase 3 and cleaved PARP.	
5-DMN	Apoptosis-inducing	HCT116 (p53 ^+/+^) and HCT116 (p53 ^−/−^); HCT116 (Bax ^+/−^) and HCT116 (Bax ^−/−^); HCT116 (p21 ^−/−^)	Apoptosis assay Annexin-V/PI	- At 15 μM, 5-DMN increased late apoptotic/necrotic cell in HCT116 (p53 ^−/−^) > HCT115 (p53 ^+/+^), suggesting the apoptotic inducing action is independent of p53	[80]
				- At 15 μM, 5-DMN increased early apoptotic cell in HCT116 (Bax ^+/−^), but not in HCT116 (Bax ^−/−^)	
	Cell cycle arrest		Cell cycle analysis - Propidium iodide staining	- At 15 μM, 5-DMN arrested cells at G2/M and G0/G1 phases in HCT116 (p53 ^+/+^) cells, but only caused G2/M phase arrest in HCT116 (p53 ^−/−^) cells	
				- G0/G1 is p53 dependent and G2/M is p53-independent	
NOB; 3′-DMN; 4′-DMN; 3′,4′-DMN	Cytotoxicity	HCT116, HT-29	MTT viability assay	- At 2.03 μM and 3.28 μM, NOB and 3′-DMN, respectively showed no significant cytotoxicity against HCT116 and HT-29	[54]
				- At 24.13 μM, 4′-DMN inhibited growth of HCT-116 by 45% and HT-29 by 33%	
				- At 12.03 μM, 3’,4’-DMN inhibited growth of HCT116 by 30% and HT-29 by 9%	
				- combination of all three NOB-metabolites inhibited growth of HCT116 by 64% and HT-29 by 62% (no significant difference to three NOB-metabolites + NOB)	
	Cell cycle arrest		Cell cycle analysis - Propidium iodide staining (24 h)	- NOB (40 μM) arrested cells at G0/G1 phase in both HCT-116 and HT-29	
				- 3′-DMN (40 μM) arrested cells at both S phase and G2/M phase in HCT-116; while arrested cells at both G0/G1 and G2/M phase in HT-29	
				- 4′-DMN (40 μM) induced a stronger effect than NOB in arresting cells at G0/G1 phase in HCT-116 and HT-29	
				- 3′,4′-DMN (20 μM) arrested cells at both S phase and G2/M phase in HCT-116; while arrested cells at both G0/G1 and G2/M phase in HT-29	
	Apoptosis inducing		Western blot	- NOB and all three NOB-metabolites cause profound increase in expression of p21^Cip1/Waf1^	
			Annexin-V/PI (48 h)	- NOB (40 μM) increased early apoptotic cell population by 3.3-fold, increased late apoptotic cell population by 4.2-fold in HCT116	
				- 3′-DMN (40 μM) increased early apoptotic cell population by 5.0-fold, increased late apoptotic cell population by 3.5-fold in HCT116	
				- 4′-DMN (40 μM) increased early apoptotic cell population by 4.9-fold, increased late apoptotic cell population by 7.1-fold in HCT116	
				- 3′,4′-DMN (20 μM) increased early apoptotic cell population by 7.6-fold, increase late apoptotic cell population by 4.5-fold in HCT116	
				-3′-DMN (40 μM) and 4’-DMN (40 μM) did not cause significant apoptosis in HT-29	
				- 3′,4′-DMN (20 μM) exhibits stronger apoptosis effect than NOB (40 μM) in HT-29	
			Western blot	- NOB (40 μM) only increased activation of caspase-9 and did not affect caspase-3 or PARP levels in HCT116	
				- NOB-metabolites increased activation of caspase-3, caspase-9 and other downstream proteins like PARP in HCT116	
NOB-Met (2.03 μM NOB: 3.28 μM 3′-DMN: 24.13 μM 4′-DMN: 12.03 μM 3′,4′-DMN	Anti-inflammatory	RAW264.7	Western Blot	- At 0.5× concentration of NOB-Met, supressed LPS-induced iNOS expression by 56.4%	[76]
				- At 1× and 2× concentration of NOB-Met, completely abrogated LPS-induced iNOS expression	
				- At ×0.5, increased expression of NQO1 by 21% as compared to LPS-treated cells	
				- At ×1, increased expression of HO-1 by 10%, increased expression of NQO1 by 34% as compared to LPS-treated cells	
				- At ×2, increased expression of HO-1 by 37%, increased expression of NQO1 by 50% as compared to LPS-treated cells	
				- Induced translocation of Nrf2	
	Cell cycle arrest	HCT116	Cell cycle analysis - Propidium iodide staining Western blot	- At 1×, induced G0/G1 phase arrest; while at 2×, induced G0/G1 and G2/M phases arrest	
				- Reduced expressions of CDK-2, CDK-4, CDK-6 and cyclin D, while increased expressions of p53 and p27	
NOB, 5-DMN	Cytotoxicity	HCT116, HT-29, COLO205	MTT viability assay	- At 40 μM, NOB significantly reduced viability of HCT116, HT-29 and COLO205 by ~20–30%	[49]
				- At >5 μM, 5-DMN significantly reduced viability of HCT116, HT-29 and COLO205	
	Apoptosis inducing		Cell cycle analysis - SubG1 quantification Western	- At 20 μM, 5-DMN increased apoptosis ratio by ~26%, while no increased in subG1 population in NOB-treated COLO205	
				- At 10 and 20 μM, significantly increased expression of cleaved PARP in COLO205	
NOB	Anti-inflammatory	Human synovial fibroblast, mouse macrophage J774A.1	ELISA	- At >4 μM, NOB inhibited PGE_2_ induced by IL-1α in human synovial fibroblast	[81]
			Western blot and qPCR	- At >16 μM, NOB reduced mRNA of COX-2 induced by IL-1α in human synovial fibroblast	
				- At 64 μM, NOB inhibited COX-2 protein expression induced by IL-1α in human synovial fibroblast	
			qPCR	- At 32 μM, NOB reduced mRNA of IL-1α, IL-1β, IL-6, TNF-α induced by LPS in J774A.1	
			Western blot	- At >16 μM, NOB reduced proMMP-1 and proMMP-3 induced by IL-1α in human synovial fibroblast	
				- At >16 μM, NOB enhanced TIMP-1 expression in response to IL-1α in human synovial fibroblast	
NOB	Anti-inflammatory	Mouse adipocyte 3T3-L1	ELISA	- At 50 and 100 μM, NOB suppressed MCP-1 secretion induced by TNF-α IN 3T3-L1 adipocytes	[82]
			Western blot	- At 50 and 100 μM, NOB reduced ERK phosphorylation in 3T3-L1 adipocytes treated with TNF-α	

IC_50_—half maximal inhibitory concentration; AP-1—activator protein-1; PI—propidium iodide; PARP—poly (ADP-ribosome) polymerase; CDK—cyclin-dependent kinase; Rb—retinoblastoma; LPS—lipopolysaccharide; iNOS—inducible NO synthase; NQO1—NAD(P)H quinone oxidoreductase 1; HO-1—heme oxygenase-1; PGE_2_—prostaglandin E2; IL—interleukin; TNF—tumor necrosis factor; TIMP-1—tissue inhibitor metalloprotease-1; MCP-1—monocyte chemoattractant protein-1.

**Table 2:** In vivo studies of NOB for colon cancer chemoprevention.

Animal Models	Treatment/Dosage	Mechanisms	Detailed Results	References
Colitis-associated colon carcinogenesis model - AOM (12 mg/kg i.p.)/1% DSS in drinking water treated male CD-1 mice (5-week-old)	AIN93G diet containing 0.05% wt NOB (20 weeks)	Cell cycle arrest	Protein expression in colonic mucosa by Western blot - Reduced levels of CDK-2, CDK-4, CDK-6, cyclin D and cyclin E - Increased levels of p21, p27 and p53	[76]
		Anti-inflammatory effects	Immunohistochemical analysis - Reduced expression of iNOS reduced by 35% when compared to the positive control Protein expression in colonic mucosa by Western blot - Increased level of HO-1 - Increased level of NQO1 - Induced translocation of level of Nrf2 transcription factor (Nuclear fraction < Cytoplasmic fraction)	
Colitis-associated colon carcinogenesis model - AOM/DSS treated AOM (12 mg/kg i.p.)/1% DSS in drinking water treated male CD-1 mice (5 week old)	AIN93G diet containing 0.05% wt NOB (20 weeks)	Inhibit AOM/DSS-induced colon carcinogenesis	- Prevented shortening of colon length, reduced the increased colon weight/length ratio - Reduced tumor incidence by 40% and tumor multiplicity by 71% - Maintained histological characteristic of normal mucosa	[54]
		Anti-proliferative effect	- Reduced PCNA-positive colonocytes by 69% in mucosal crypts	
		Apoptosis-inducing effect	- Increased cleaved caspase-3 positive cells by 2.3-fold in colonic tumor	
		Anti-inflammatory effects	- Reduced levels of proinflammatory cytokines - ELISA showed reduction of TNF-α by 51%, IL-1ß by 92% and IL-6 by 69% compared - qRT-PCR analysis showed reduction of TNF-α by 65%, IL-1ß by 69% and IL-6 by 45%	
Colon carcinogenesis model - AOM (15 mg/kg i.p.) treated male *db*/*db* mice	Diet containing 100 ppm NOB (0.1% wt) (10 weeks)	Inhibit AOM induced colon carcinogenesis	- Reduced frequency of preneoplastic lesions (colonic aberrant crypt foci (ACF) and β-catenin-accumulated crypts (BCAC)) - Reduced incidence of ACF by 68-91% and BCAC by 64–71% - Reduced PCNA-labeling index in ACF by 21% and BCAC by 19%	[83]
Colon carcinogenesis model - AOM (10 mg/kg i.p.)/1% DSS in drinking water treated male CD-1 mice	Diet containing 100 ppm NOB (0.1% wt) (for 17 weeks)	Inhibit AOM/DSS-induced colon carcinogenesis	- Suppressed incidence of neoplasms (adenoma and adenocarcinoma), lowered multiplicity of tumor	[84]
		Inhibit leptin-induced colon carcinogenesis		
			- Suppressed serum levels of leptin by 75–84%	
Colon carcinogenesis model - AOM (20 mg/kg s.c.) treated male F344 rats	Diet containing NOB (0.01% wt and 0.05% wt) (34 weeks)	Inhibit AOM induced colon carcinogenesis	- Reduced incidence and multiplicity of colonic adenocarcinoma	[74]
		Anti-proliferative effect		
			- Increased apoptosis index of adenocarcinoma	
		Anti-inflammatory effect		
			- Reduced level of PGE_2_ in colonic adenocarcinoma and surrounding mucosa	
Colon carcinogenesis model - AOM (20 mg/kg s.c.) treated male F344 rats	Diet containing NOB (0.01% wt and 0.05% wt) (5 weeks)	Inhibit AOM-induced colon carcinogenesis	- Reduced the frequency of colonic aberrant crypt foci formation - Reduced number of ACF in proximal, middle and distal colon	[41]
		Anti-proliferative effect		
			- Reduced MIB-5 labeling index of ACF but not of normal colonic crypts	
		Anti-inflammatory effect		
			- Reduced level of PGE_2_ in colonic mucosa	
Colon carcinogenesis model - PhIP hydrochloride (100 mg/kg i.g.) treated F344 male rats (twice/week for 10 weeks)	Diet containing NOB (0.05% wt.) (50 weeks)	Inhibit PhIP-induced ACF in transverse colon	- Reduced the total colonic ACF indices in transverse colon	[75]
Colorectal cancer xenograft mouse model - COLO205 cells s.c.	NOB 100 mg/kg i.p. daily for 3 weeks 5-DMN 50 mg/kg and 100 mg/kg i.p. daily for 3 weeks	Anti-tumor effect	- NOB reduced tumor size and weight but not significant as compared to control - 5-DMN reduced tumor size and weight significantly as compared to control	[49]
		Autophagy induction	- 5-DMN increased LC3 expression	
		Anti-inflammatory effect		
			- 5-DMN increased p53 expression - 5-DMN reduced COX-2 expression	
		Anti-angiogenesis		
			- 5-DMN reduced VEGF expression	

AOM—azoxymethane; DSS—dextran sulfate sodium; i.p.—intraperitoneal injection; s.c.—subcutaneous injection; i.g.—intragastric administration; PCNA—proliferating cell nuclear antigen; ACF—aberrant crypt foci; BCAC—β-catenin-accumulated crypts; PhIP—1-Methyl-6-phenyl-1H-imidazo[4,5-b]pyridin-2-amine; LC3—microtubule-associated protein light chain 3; VEGF—vascular endothelial growth factor.
